# Amazonian Bacuri (*Platonia insignis* Mart.) Fruit Waste Valorisation Using Response Surface Methodology

**DOI:** 10.3390/biom11121767

**Published:** 2021-11-25

**Authors:** Klenicy K. L. Yamaguchi, David S. Dias, Carlos Victor Lamarão, Karen F. A. Castelo, Max S. Lima, Ananda S. Antonio, Attilio Converti, Emerson S. Lima, Valdir F. Veiga-Junior

**Affiliations:** 1 Institute of Health and Biotechnology, Federal University of Amazonas, Coari 69460-000, Brazil; 2 Institute of Mathematical and Computer Sciences, University of São Paulo, São Carlos 13566-590, Brazil; souzaddias@gmail.com; 3 Agricultural Products Technology Laboratory, Faculty of Agricultural Science, Federal University of Amazonas, Manaus 69080-900, Brazil; victorlamarao@yahoo.com.br; 4 Chemistry Department, Institute of Exact Sciences, Federal University of Amazonas, Manaus 69077-000, Brazil; karen.falves@gmail.com (K.F.A.C.); maxlima@ufam.edu.br (M.S.L.); 5 Center for Forensic Analysis, Laboratory for the Support of Technological Development, Chemistry Institute, Federal University of Rio de Janeiro (NAF–LADETEC/IQ–UFRJ), Rio de Janeiro 21941-598, Brazil; ananda.antonio@gmail.com; 6 Department of Civil, Chemical and Environmental Engineering, Pole of Chemical Engineering, University of Genoa, I-16145 Genoa, Italy; converti@unige.it; 7 Faculty of Pharmaceutical Sciences, Federal University of Amazonas, Manaus 69080-900, Brazil; 8 Chemical Engineering Section, Military Institute of Engineering, Rio de Janeiro 22290-270, Brazil

**Keywords:** bacuri, Amazonia, *Platonia insignis*, antioxidants, prenylated benzophenones, food residues, biorefinery, response surface methodology, radical scavenging capacity, green chemistry

## Abstract

Bacuri (*Platonia insignis* Mart) is a species from the Clusiaceae genus. Its fruit pulp is commonly used in South America in several food products, such as beverages, ice cream and candies. Only the pulp of the fruit is used, and the peels and seeds are considered waste from these industries. As a trioxygenated xanthone source, this species is of high interest for bioproduct development. This work evaluated the mesocarp and epicarp of bacuri fruits through different extraction methods and experimental conditions (pH, temperature and solvent) in order to determine the most effective method for converting this agro-industrial waste in a value-added bioproduct. Open-column procedures and HPLC and NMR experiments were performed to evaluate the chemical composition of the extracts, along with total phenols, total flavonoids and antioxidant activities (sequestration of the DPPH and ABTS radicals). A factorial design and response surface methodology were used. The best extraction conditions of substances with antioxidant properties were maceration at 50 °C with 100% ethanol as solvent for mesocarp extracts, and acidic sonication in 100% ethanol for epicarp extracts, with an excellent phenolic profile and antioxidant capacities. The main compounds isolated were the prenylated benzophenones garcinielliptone FC (epicarp) and 30-*epi*-cambogin (mesocarp). This is the first study analysing the performance of extraction methods within bacuri agro-industrial waste. Results demonstrated that shells and seeds of bacuri can be used as phenolic-rich bioproducts obtained by a simple extraction method, increasing the value chain of this fruit.

## 1. Introduction

Bacuri is a South American species of Clusiaceae named *Platonia insignis* Mart. It is found from Paraguay to Suriname and is widely distributed in the Amazon and Cerrado biomes [1]. The fruit is famous for its pleasant and bittersweet taste, being largely consumed in food products such as beverages, ice cream and candies, for its sensorial attributes and for its remarkable biological properties [2,3]. Despite its commercial potential, its production still relies on extractivism, with an annual production of 3,061 tonnes of fresh fruit in 2017 [4,5]. The pulp used in the production of food comprises only 26% of the fresh fruit’s weight, with the other 58% of the weight considered waste [5,6,7]. This significant waste of biomass could be used to improve the local bioeconomy and develop new bioproducts, as several studies reported that *P. insignis*’s volatile fractions and the polar extracts of its seeds are rich in antioxidant, anti-inflammatory, anticonvulsant, antiparasitic, hypotensive and immunoregulatory compounds [1,2,3,8]. Moreover, the chemical profile of the bioproduct highlights terpenes and phenolic compounds as its main bioactive compounds [9,10].

Phenolic compounds are of great interest to industry due to their antioxidant potential. The food and cosmetics industries, in particular, have been using them as food preservatives and active agents for antiaging skin creams, respectively. In addition, they are also used in so-called healthy foods as well as the treatment of diseases resulting from the action of free radicals [3,11]. Fruit peels and seeds, which are generally not used in industrial processes, may be promising sources of phenolic substances. However, there are still few initiatives to develop processes so that industries can use this waste. For instance, none of the published research of *P. insignis* has aimed to determine better processing methods to add value to its agro-industrial waste. The studies on Amazonian biodiversity still rely mainly on chemical characterization [12,13], searching for patterns [14] or drug development based on traditional knowledge [15].

The optimization of extraction processes of biologically active substances is a procedure that has become considerably more common in recent years [16,17,18], and the search for sustainable, efficient and selective extraction methods is one of the main goals of industry. Generally, the effectiveness of the extraction of phenolic compounds from natural products is influenced by multiple factors, such as extraction temperature, time, water-to-raw-material ratio, pressure, and type of solvent. In related studies, results are commonly processed with the aid of statistical tools, such as response surface methodology [19,20,21,22], which allow for the selection of optimal conditions under which it is possible to recover the maximum number of compounds of interest. There are few reports on the optimization of the extraction of crude phenolic compounds from *P. insignis* and their antioxidant activities [8,10,11]. Therefore, due the growing consumption of this fruit, its described biological properties and the number of bioactive substances in its extract, here it is proposed to determine the best extraction method for antioxidant substances from bacuri shell (mesocarp and epicarp) using the response surface methodology in order to obtain a valuable bioproduct.

## 2. Materials and Methods

### 2.1. Chemicals and Reagents

Trolox (6-hydroxy-2,5,7,8-tetramethylchroman-2-caboxylic acid) (Sigma-Aldrich, St. Louis, MO, USA), DPPH (2,2-diphenyl-picrylhydrazyl) (Sigma-Aldrich, St. Louis, MO, USA), gallic acid (Sigma-Aldrich, St. Louis, MO, USA), quercetin (Sigma-Aldrich, St. Louis, MO, USA), DMSO (Sigma-Aldrich, St. Louis, MO, USA), TMS (Sigma-Aldrich, St. Louis, MO, USA), CH_3_OD (Sigma-Aldrich, St. Louis, MO, USA), and CDCl3 (Sigma-Aldrich, St. Louis, MO, USA), and Folin–Ciocalteu reagent (Sigma–Aldrich Chemie, Steinheim, Germany) was used. Ethanol (Merck, Darmstadt, Germany) and methanol (Merck, Darmstadt, Germany), the extraction solvents, were HPLC grade.

### 2.2. Biological Samples

Shells from *P. insignis* were provided by the Fazenda Bacuri, located in Bragança city, Pará state, Brazil. A voucher sample was deposited at UFAM herbarium and the biological sampling was registered on the National System of Genetic Resource Management and Associated Traditional Knowledge (SISGEN), in accordance with Brazilian legislation regarding biodiversity scientific exploitation. Shells were manually removed from ripe fruits and divided in mesocarp (PM) and epicarp (PE). Both shell parts were dried in an air circulation oven at 40 °C for 48 h, before being crushed in a knife mill.

### 2.3. Extraction Procedures and Analysis

Evaluation of the extraction technique and solvent was done through a set of experiments carried out according to a 2^4^-factorial experimental design. The levels used for the extraction technique were cold maceration (CM), hot maceration (HM), neutral sonication (NS), and acid sonication (AS). CM and HM required the sample to be submersed in the extraction solvent for 24 h at room temperature (25.0 ± 0.5 °C) and for 2 h at 50 °C, respectively. Both NS and AS were ultrasound-assisted extractions performed in a bath-type ultrasound apparatus (Q1.8/40A model, 40kHz frequency, Ultronique, Brazil) for 20 min at room temperature (25 ± 0.5 °C), with the only difference being the addition of 50 µL of hydrochloric acid in the extraction solvent used in the latter method. Recent optimization studies regarding phenolic extraction from botanic matrices indicated that acidity solvents can increase extraction performance, depending on the phenolic compounds profile (e.g., flavonoids, anthocyanins, and others) [23,24,25]. In all extraction methods, a sample-to-solvent ratio of 1:10 (g/mL) was used. The extraction solvent levels tested were different concentrations of ethanol in the aqueous solvent, namely 100%, 80%, 50% and 20%. After each experiment, the obtained extract was dried using a rotary vacuum evaporator (801 model, Fisatom, Brazil) and a desiccator. The dried extracts were stored under refrigeration prior to analysis. The response variables (*Y_ijk_*) used were yield of extraction (%), total phenolic content (%), total flavonoid content (%), antioxidant capacity of the 2,2′-azino-bis (3-ethylbenzothiazoline-6-sulfonic acid) (ABTS) assay expressed as scavenging effect (%) and antioxidant capacity of the DPPH^•^ assay expressed as half maximal inhibitory concentration (IC_50_, %). The structural model used for the factorial design is represented by Equation (1): DPPH

(1) $$Y_{ijk}=\mu +\tau_{j}+\beta_{k}+\gamma_{jk}+\epsilon_{ijk}$$

where *μ =* (*μR*, *μFe*, *μFl*) is the global mean vector, *τ_j_* *=* (*τ_j_R*, *τ_j_Fe*, *τ_j_Fl*) is the effect of the extraction method (*j*), *β_k_* *=* (*β_k_R*, *β_k_Fe*, *β_k_Fl*) is the effect of the type of solvent (*k*), *γ_jk_ =* (*γ_jk_R*, *γ_jk_Fe*, *γ_jk_Fl*) is the effect of interaction between extraction method and type of solvent and *Є_ijk_* is the random error.

Analysis of the phenolic compound extraction was performed by applying multivariate analysis of variance (MANOVA) to the results of the experimental designs [26]. Statistical analysis was performed using the R. 2.14.0 statistical software (Free Software Foundation, Boston, MA, USA). The response surface methodology was used to identify the best conditions to obtain the active extracts, while the comparison of means and the factor levels (method, and solvent-solvent method) as independent variables was performed by analysis of variance (ANOVA) followed by a Tukey test.

The crude extraction yield was determined using the gravimetric method, taking into account the dry weight of the extract and the weight of botanical material used during extraction. The ethanolic extracts were fractionated with less polar solvents by liquid–liquid extraction, followed by open-column chromatography. Identification procedures were performed by mass spectrometry (LCQ Fleet, from Thermo Scientific, operating with APCI and ESI modes, samples inserted directly, in HPLC methanol) and nuclear magnetic resonance (Advance III HD 11.75 Tesla, from Bruker, using TMS as internal standard and CD_3_OD and CDCl_3_ as solvents).

### 2.4. Response Variable Determination

The extraction methods and optimization of the extraction solvent were performed by the evaluation of five response variables: the crude extraction yield, total phenolic content, total flavonoid content, and the antioxidant activity capacity based on DPPH and ABTS assay.

#### 2.4.1. Total Phenolic Content

The quantification of phenolic compounds was performed according to the Folin-Ciocalteu method described by Singleton and Rossi [27]. The extracts (10 µL) were mixed with 50 µL of Folin–Ciocalteu reagent for 8 min, and then 240 µL of sodium carbonate were added. After incubation at room temperature for 3 min, the absorbance of the reaction mixture was measured at 715 nm against a methanol blank using a microplate reader (DTX 800 multimode detector, UV/Vis spectrophotometer, Beckman Coulter, Brea, CA, USA). Gallic acid was used as a standard. The experiments were performed in triplicate. Data of the total phenolic content in the dry matter were expressed as mean percentages.

#### 2.4.2. Total Flavonoids

The quantification of total flavonoids was performed by the colorimetric method by Chang et al. [28], adapted to microplate. The extract solution (30 µg of 1:10 g.mL^−1^ solution) in dimethyl sulfoxide (DMSO) was individually mixed with 90 µL of ethanol, 6 µL of 10% aluminium chloride, 6 µL of 1.0 M potassium acetate and 168 µL of distilled water. The resulting mixture was maintained at room temperature for 30 min, and its absorbance measured at 405 nm with the microplate reader described above. All samples were analysed in triplicates. Data of the total flavonoid content in the dry matter were expressed as mean percentages.

#### 2.4.3. ABTS Radical Scavenging Assay

ABTS radical cation (ABTS^•+^) scavenging activity was measured by the method reported by Re et al. with minor modification [29]. The ABTS^•+^ solution was prepared by combining the reaction of the ABTS stock solution with 2.45 mM potassium persulfate and allowing the mixture to stand in the dark at room temperature for 12–16 h before use. The ABTS^•+^ solution was diluted to the absorbance of 0.70 ± 0.02 and stored for offline and online assays. For analysis, 30 µL of diluted extracts at different concentrations (1–100 µg mL^−1^) were added to 270 µL of ABTS^•+^ solution and left in the dark at room temperature for 15 min. The absorbance was measured at 630 nm with the above microplate reader. All determinations were carried out in triplicate for each concentration of either standard or samples. The percentage inhibition of absorbance at 630 nm was calculated and plotted as a function of concentration of antioxidants and Trolox (standard reference) using Equation (2).

(2) $$\mathrm{Scavenging}\;\mathrm{effect}\;\left(\%\right)=\left[\frac{\left(Abs_{control}-Abs_{sample}\right)}{Abs_{control}}\right]\times 100$$

where *Abs_control_* is the absorbance of the control solution without sample.

#### 2.4.4. DPPH Radical Scavenging Assay

DPPH^•^ radical scavenging activity was determined according to the method described by Molyneux with slight modification [30]. A total of 30 μL of extracts were diluted in DMSO and mixed with 270 μL of DPPH in ethanol on a 96-well plate. After keeping the plate in the dark for 30 min, the absorbance of the solution was measured at 517 nm in the same microplate as above. Blanks (DMSO) and standards (quercetin solutions in DMSO) were analysed simultaneously. Extracts were first tested only at the concentration of 100 µg mL^−1^, and those showing good evidence of antioxidant activity were tested over a concentration range to determine the IC_50_. The IC_50_ was calculated using Equation (3):

(3) $${\mathrm{IC}}_{50}\;\left(\%\right)=\left[\frac{\left(Abs_{blank}-Abs_{sample}\right)}{Abs_{blank}}\right]\times 100$$

where *Abs_blank_* is the absorbance of the control reaction (containing all reagents except the test compound).

## 3. Results and Discussion

The development of extraction procedures with less toxic solvents to the environment is one of the green chemistry postulates. The solvents most used in the extraction of bioactive compounds are methanol, ethanol, acetone, ethyl acetate and water [23,31]. In order to comply with the green chemistry postulates, ethanol/water mixtures were selected as solvents in this study. The results of tests carried out with different extraction methods at different ethanol percentages in water are summarized in Table 1 for both the mesocarp and epicarp of bacuri (*P. insignis*).

### 3.1. Crude Extraction Yield

The crude extraction yield (CEY) was significantly influenced by the solvent-water ratio. Mesocarp samples (MS) presented a higher CEY (36.63%) than any epicarp samples, highlighting the different chemical compositions of each fruit part. For MS, most of the tested methods presented an increasing pattern of CEY until 100% ethanol was used as the extraction solvent. An exception was observed for acid sonication, in which the highest CEY was obtained with 80% ethanol (Table 1). Methods applied to epicarp samples (ES) were distinctively influenced by the percentage of ethanol in the extraction solvent. Maceration methods presented an increasing CEY until 80% ethanol, while ultrasonic-based methods decreased CEY linearly as more ethanol was used in the extraction solvent (Table 1). The different behaviour observed in sonication methods may be associated with the increase in the extraction solvent viscosity caused by the addition of ethanol, which reduces the cavitation process within the solvent [31]. Of the tested methods, the maceration techniques showed the highest extraction yields for both sample types. The CEY of maceration methods was also significantly influenced by the extraction temperature of the extraction method. When 80% ethanol (which was the solvent that produced the highest CEY of ES) was used, the highest temperature increased CEY by approximately 20% for both sample types (Table 1).

### 3.2. Chemical Characterization

**Figure 1:**
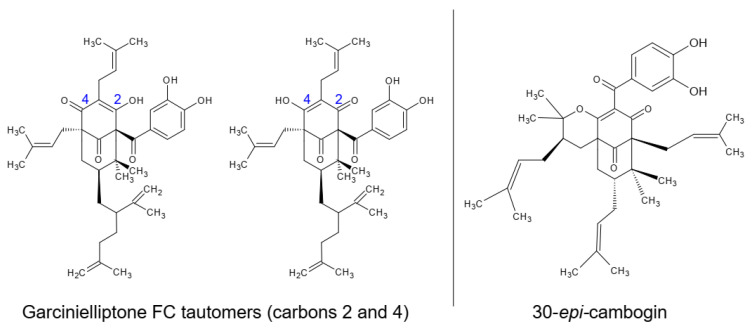
Identified prenylated benzophenones on epicarp (garcinielliptone FC tautomers) and mesocarp (30-*epi*-cambogin).

The ethanolic extracts of epicarp and mesocarp were partitioned with medium polarity solvents (dichloromethane and ethyl acetate) and fractionated using chromatographic techniques to isolate the major compounds of these residues. Different open-column chromatography fractionations with silica gel and low-to-medium polarity solvents were performed, which returned the isolation of two substances, with one returned from each bacuri fruit part. The substances were evaluated by mass spectrometry and nuclear magnetic resonance, showing similar structures and the same low resolution molecular mass: prenylated benzophenones with mass 602 u.m.a. In samples from the epicarp, the tautomers from garcinielliptone (Figure 1) were detected using APCI mass spectrometry with *m*/*z* 603 (positive mode) and *m*/*z* 601 (negative mode). This is a common substance in the Clusiaceae family and *Garcinia* genus. The confirmation was performed by NMR experiments with ^1^H, ^13^C, HSQC and HMBC, the results of which were compared with literature data obtained in CDCl_3_ [32,33], together with the analysis of the fragments *m/z* 465 and *m*/*z* 409 observed by MS^n^ from *m*/*z* 601 ionized in APCI (-) [34].

From the mesocarp, another prenylated benzophenone, 30-*epi*-cambogin, was isolated. With the same molecular mass, 602 u.m.a., MS^n^ from *m/z* 601 ionized in APCI (-) allowed the observation of fragments such as *m/z* 136, which is typical from phloroglucinol units. The confirmation was also performed by ^1^H, ^13^C, HSQC and HMBC NMR experiments, the results of which were compared with literature data (obtained in CD_3_OD) [35].

### 3.3. Evaluation of Phenolic Composition and Antioxidant Capacity

Phenolic and flavonoid compounds are known antioxidants agents that are capable of inhibiting the oxidation of low-density lipoproteins and stabilizing unstable radicals. Therefore, their content can be used to assess the nutritional value and biological properties of different matrices [1,4,5]. To evaluate the effects of the extraction methods and solvent compositions on the total flavonoid content, total phenolic content and antioxidant capacity, it was first evaluated whether these response variables have high linear correlation (nominal correlation higher than 0.800) using multivariate analysis (MANOVA). High correlation can provide insight if response variables are independent from each other and also highlight which statistical methods is better suited for analysing the data. Correlation between each response variable was weak within both sample types during MANOVA (Table 2), indicating the need for individual analysis instead of multivariate. Thus, an individual factorial experimental design was adopted for each of these response variables.

Since the correlation of response variables by multivariate statistic was weak (Table 2), univariate analysis of variance (ANOVA) was conducted. ANOVA demonstrated that within each response variable there was a significant interaction effect between the extraction method and solvent composition, with *p*-value < 0.0001, indicating the possibility to screening method performance through response surface methods.

**Figure 2:**
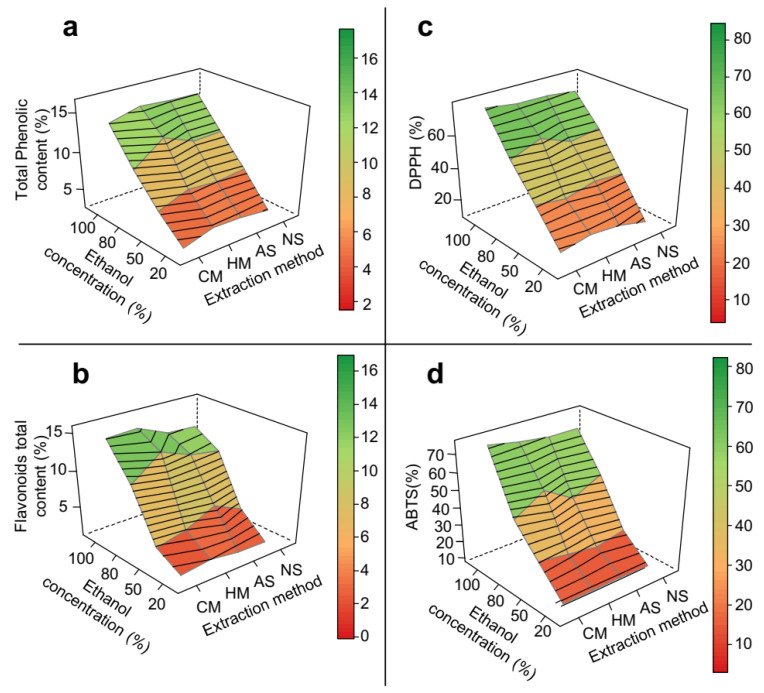
3D response surface graph of the optimization of phenolic extraction from *P. insignis* mesocarp. (**a**) Total phenolic, (**b**) Total flavonoid, (**c**) DPPH, and (**d**) ABTS. CM = cold maceration; HM = hot maceration; AS = acid sonication; NS = neutral sonication; DPPH = Antioxidant capacity by DDPH assay; ABTS = Antioxidant capacity by DDPH assay.

Response surface methodology demonstrated that the extraction method, temperature and pH did not significantly affect the results of mesocarp extraction, producing plane-like surfaces (Figure 2). Total phenolic content, total flavonoid content, and antioxidant activities of DPPH and ABTS assays exhibited qualitatively the same pattern observed for the crude extraction yield, with almost all linearly increasing as ethanol proportion in the solvent increased from 20 to 100%. These increases were 4.7-fold (Figure 2a), 13-fold (Figure 2b), 5-fold (Figure 2c) and 7-fold (Figure 2d), respectively, which suggests that these classes of compounds are linked to the matrix through different types of bonds. Such increases can be related, in general, to the ability of ethanol to induce disruption of vegetable cell membranes, thereby enhancing solvent permeability into the solid matrix [36]. The more effective the extraction of phenolic compounds and flavonoids, the stronger the antioxidant activity almost regardless of their detection method.

**Figure 3:**
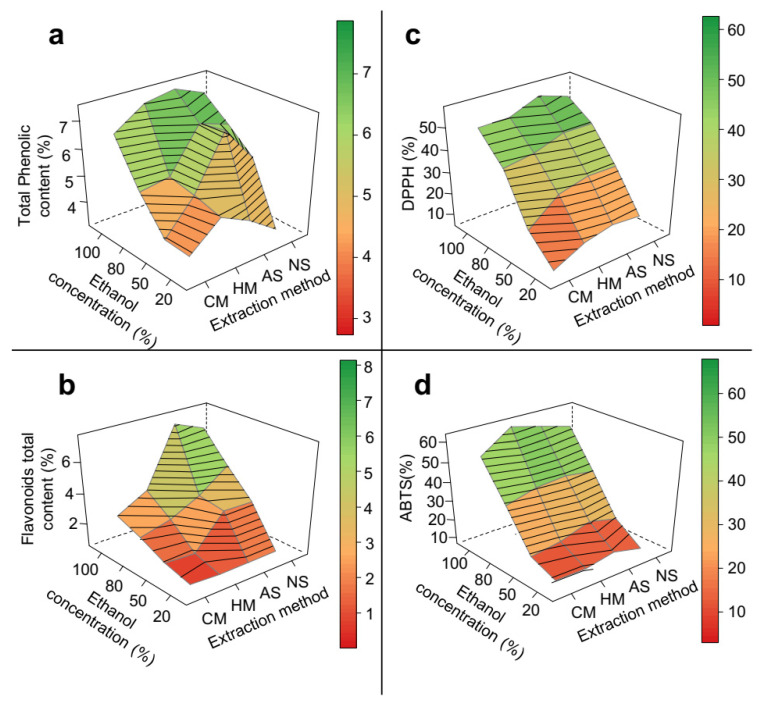
3D response surface graph of the optimization of phenolic extraction from *P. insignis* epicarp. (**a**) Total phenolic, (**b**) Total flavonoid, (**c**) DPPH, and (**d**) ABTS. CM = cold maceration; HM = hot maceration; AS = acid sonication; NS = neutral sonication; DPPH = Antioxidant capacity by DDPH assay; ABTS = Antioxidant capacity by DDPH assay.

In epicarp samples, the type of extraction method had a significant impact on the total content of both phenolic compounds (Figure 3a) and flavonoids (Figure 3b). Ultrasound methods produced higher yields of bioactive compounds compared to maceration methods. The ultrasound-assisted methods had as a major extraction mechanism, the sonication phenomenon, which produced soundwave shocks capable of disrupting plant cells, and thus facilitated the diffusion of the solvent into the plant matrix [31]. Even though the effects of the considered factors on the radical scavenging activity of epicarp samples were in general not statistically significant (Table 1), it was possible to observe an increase in the ABTS radical inhibition when ultrasound methods were applied (Figure 3d).

The different results obtained with AS and NS indicate that a change in the pH of the extraction solution interfered with ES extraction performance (Figure 3). In contrast to the extraction yield, there was a significant increase (approximately 13%) in the effectiveness of phenolic compound and flavonoid extraction when a more acidic medium was used for AS, but not for NS (Table 1, Figure 3). Similar results have been reported in the optimization of phenolics extraction from other plant matrices such as *Citrus reticulata* [37]. In general, the acidity increased the flavonoid content by inducing the cleavage of their bond with proteins and favoring their occurrence in medium as protonated molecules [23,37].

As expected, for both PS and ES, a rise in the extraction temperature improved the results of all the response variable (Figure 2 and Figure 3). An increase in temperature is known to favour the extraction of some classes of phenolic compounds by (a) increasing their solubility and diffusion rate, (b) reducing the solvent surface tension and viscosity and (c) increasing ethanol reactivity due to a decrease in its dielectric constant [21,23,31,37]. However, the use of high temperatures in phenolic extraction must be closely monitored, given that temperatures that are too high temperatures can expose the bioactive compounds to the risk of degradation [24]. Previous optimization studies on the extraction of phenolics and antioxidant compounds from *Theobroma cocoa* shells, *Olea europaeae*, green tea, and *Citrus* species have shown the highest recovery rates at temperatures ranging from 43 °C to 70 °C [18,20,38,39,40].

It is worth mentioning that using all extraction methods, an increased ethanol fraction in the solvent improved the results of all response variables for both PS and ES extraction. There are several features of the extraction solvent that can influence its efficacy. Due to differences in polarity, intermolecular interactions and stereochemistry, there is no suitable solvent extraction system to recover specific classes of natural antioxidants, and the efficacy of a specific solvent will depend on the matrix and its chemical profile. For instance, more nonpolar flavonoids will require less polar extraction solvents [23,37,41].

It is believed that the type of solvent, temperature and pH can determine the quantity of phenolic compounds to be extracted [23]. However, it is not possible to find data in the literature that relate these three parameters with the aim of identifying an ideal standard condition. Since each raw material has its own characteristics that depend on the matrix chemical composition, the identification of the optimal extraction parameters is of paramount important for successful recovery of bioactive compounds.

## 4. Conclusions

Statistical methods such as the response surface methodology are widely used as tools to optimize processes for the recovery of various chemical components from different fruits. Although some variables investigated in this study, such as the antioxidant activity in absolute ethanol, did not show statistical differences in their results, this tool allowed for the analysis of the whole dataset successfully. From the results of extraction yield, content of total phenolic compounds and total flavonoids, and antioxidant activity by both DPPH and ABTS assays, we identified hot maceration with 100% ethanol and sonication aided by hydrochloric acid added to 100% ethanol as the optimal extraction method for bacuri mesocarp and epicarp, respectively.

## Figures and Tables

**Table 1:** Results of extractions carried out with different methods at different ethanol percentages in water on the mesocarp and epicarp of bacuri.

Sample	Extraction Method	% of Ethanol	Crude Extraction Yield (%) ± SD	Total Phenolic Compounds (% *w*/*w* ± SD)	Total Flavonoids (% *w*/*w* ± SD)	Antioxidant Capacity Assays (% of Inhibition)	
						DPPH	ABTS
Mesocarp	Cold maceration	100	30.18 ± 1.45	14.60 ± 0.42	14.51 ± 0.24	77.34 ± 2.13	74.09 ± 1.48 **
		80	28.22 ± 1.28	9.59 ± 0.50	10.06 ± 0.36	58.16 ± 2.29	44.12 ± 3.21
		50	22.16 ± 1.52	5.23 ± 0.30	2.54 ± 0.19	33.91 ± 1.78	25.80 ± 1.47
		20	13.49 ± 0.61	2.64 ± 0.16	1.12 ± 0.08	15.14 ± 0.76	10.06 ± 0.42
	Hot maceration	100	36.63 ± 0.55	15.80 ± 0.83	15.05 ± 0.80	74.81 ± 1.25	73.31 ± 1.21 **
		80	31.95 ± 2.03	12.34 ± 0.04	12.50 ± 0.42	61.10 ± 1.17	48.91 ± 3.37
		50	27.62 ± 0.26	6.86 ± 0.30	3.72 ± 0.19	33.75 ± 1.72	24.26 ± 1.18
		20	20.49 ± 0.85	3.64 ± 0.14	1.64 ± 0.10	16.72 ± 0.78	9.95 ± 0.31
	Neutral sonication	100	27.88 ± 1.36	14.12 ± 0.72	12.93 ± 0.42	72.78 ± 1.16	70.38 ± 1.55
		80	25.62 ± 0.62	11.50 ± 0.48	11.26 ± 0.99	54.30 ± 1.12	51.05 ± 2.50
		50	24.26 ± 0.25	6.42 ± 0.59	4.29 ± 0.15	34.26 ± 1.71	24.27 ± 1.23
		20	19.84 ± 0.73	2.86 ± 0.23	0.97 ± 0.04	10.18 ± 0.94	8.59 ± 0.66
	Acid sonication	100	16.98 ± 0.69	14.31 ± 0.53	13.95 ± 0.62	74.14 ± 1.31	73.49 ± 2.82 **
		80	18.25 ± 1.34	10.22 ± 0.54	10.87 ± 0.48	54.61 ± 4.20	39.18 ± 1.80
		50	14.95 ± 0.61	6.38 ± 0.46	6.18 ± 0.41	35.02 ± 1.42	20.48 ± 1.10
		20	11.70 ± 0.49	3.58 ± 0.19	1.06 ± 0.03	14.96 ± 1.02	10.19 ± 0.41
Epicarp	Cold maceration	100	10.90 ± 0.26	6.51 ± 0.41	2.85 ± 0.15	52.14 ± 2.23	54.89 ± 1.92
		80	**17.32 ± 0.56**	5.47 ± 0.25	2.03 ± 0.11	34.56 ± 1.78	32.90 ± 1.08
		50	15.79 ± 0.76	3.56 ± 0.22	1.04 ± 0.09	15.47 ± 1.00	14.69 ± 0.96
		20	8.85 ± 0.29	3.57 ± 0.18	0.68 ± 0.04	5.07 ± 0.39	9.08 ± 0.59
	Hot maceration	100	12.69 ± 0.53	7.02 ± 0.31	3.97 ± 0.23	53.36 ± 2.37	58.58 ± 2.62
		80	14.47 ± 0.83	5.15 ± 0.37	2.37 ± 0.16	38.84 ± 1.94	38.87 ± 0.42
		50	13.38 ± 0.58	4.70 ± 0.26	1.09 ± 0.09	25.05 ± 1.54	17.15 ± 1.06
		20	10.66 ± 0.59	4.40 ± 0.27	0.61 ± 0.04	13.08 ± 1.03	9.12 ± 0.44
	Neutral sonication	100	4.84 ± 0.23	7.14 ± 0.39	**6.95 ± 0.30**	53.77 ± 1.75	56.44 ± 0.36
		80	7.80 ± 0.38	6.24 ± 0.23	4.92 ± 0.20	46.00 ± 2.33	39.99 ± 1.59
		50	10.94 ± 0.69	5.35 ± 0.29	2.99 ± 0.16	30.06 ± 2.35	18.17 ± 0.68
		20	13.73 ± 0.74	3.27 ± 0.15	0.57 ± 0.04	12.23 ± 0.71	7.81 ± 0.54
	Acid sonication	100	6.40 ± 0.39	**7.22 ± 0.16**	6.51 ± 0.39	**55.91 ± 0.86**	**60.23 ± 2.52**
		80	7.97 ± 0.07	6.62 ± 0.31	3.58 ± 0.25	42.63 ± 2.89	41.75 ± 2.48
		50	10.72 ± 0.32	6.58 ± 0.32	2.92 ± 0.25	30.52 ± 0.75	22.08 ± 1.23
		20	13.19 ± 0.87	4.02 ± 0.08	0.59 ± 0.03	14.65 ± 0.93	11.00 ± 0.84

SD = standard deviation ** Not statistically significant difference. The highest values for each response are written in bold.

**Table 2:** Multivariate analysis correlation matrix of the response variables screened.

	Mesocarp Samples					Epicarp Samples				
Response-variables	TFC	TPC	CEY	DPPH	ABTS	TFC	TPC	CEY	DPPH	ABTS
TFC	1.0000	−0.0104	0.1124	0.2391	−0.0035	1.0000	−0.0021	0.1067	0.1312	0.2697
TPC	−0.0104	1.0000	−0.3430	−0.0510	0.0334	−0.0021	1.0000	0.1552	−0.1947	0.3763
CEY	0.1124	−0.3430	1.0000	−0.0862	0.0992	0.1067	0.1552	1.0000	0.0352	0.1225
DPPH	0.2391	−0.0510	−0.0862	1.0000	−0.0684	0.1312	−0.1947	0.0352	1.0000	0.0299
ABTS	−0.0035	0.0334	0.0992	−0.0684	1.0000	0.2697	0.3763	0.1225	0.0299	1.0000

TPC = Total phenolic content; TFC = Total flavonoid content; CEY = Crude extraction yield; DPPH = Antioxidant capacity by DDPH assay; ABTS = Antioxidant capacity by DDPH assay.
